# Neural Correlates of Familiarity in Music Listening: A Systematic Review and a Neuroimaging Meta-Analysis

**DOI:** 10.3389/fnins.2018.00686

**Published:** 2018-10-05

**Authors:** Carina Freitas, Enrica Manzato, Alessandra Burini, Margot J. Taylor, Jason P. Lerch, Evdokia Anagnostou

**Affiliations:** ^1^Faculty of Medicine, Institute of Medical Science, University of Toronto, Toronto, ON, Canada; ^2^Bloorview Research Institute, Holland Bloorview Kids Rehabilitation Hospital, Toronto, ON, Canada; ^3^Sant'Anna School of Advanced Studies, Pisa, Italy; ^4^Department of Diagnostic Imaging, Hospital for Sick Children, Toronto, ON, Canada; ^5^Department of Psychology, University of Toronto, Toronto, ON, Canada; ^6^Neuroscience & Mental Health Program, Hospital for Sick Children Research Institute, Toronto, ON, Canada; ^7^Mouse Imaging Centre, Hospital for Sick Children, Toronto, ON, Canada; ^8^Department of Medical Biophysics, University of Toronto, Toronto, ON, Canada; ^9^Department of Pediatrics, University of Toronto, Toronto, ON, Canada

**Keywords:** music, familiarity, fMRI, PET, meta-analysis, activation likelihood estimation

## Abstract

Familiarity in music has been reported as an important factor modulating emotional and hedonic responses in the brain. Familiarity and repetition may increase the liking of a piece of music, thus inducing positive emotions. Neuroimaging studies have focused on identifying the brain regions involved in the processing of familiar and unfamiliar musical stimuli. However, the use of different modalities and experimental designs has led to discrepant results and it is not clear which areas of the brain are most reliably engaged when listening to familiar and unfamiliar musical excerpts. In the present study, we conducted a systematic review from three databases (Medline, PsychoINFO, and Embase) using the keywords (recognition OR familiar OR familiarity OR exposure effect OR repetition) AND (music OR song) AND (brain OR brains OR neuroimaging OR functional Magnetic Resonance Imaging OR Position Emission Tomography OR Electroencephalography OR Event Related Potential OR Magnetoencephalography). Of the 704 titles identified, 23 neuroimaging studies met our inclusion criteria for the systematic review. After removing studies providing insufficient information or contrasts, 11 studies (involving 212 participants) qualified for the meta-analysis using the activation likelihood estimation (ALE) approach. Our results did not find significant peak activations consistently across included studies. Using a less conservative approach (*p* < 0.001, uncorrected for multiple comparisons) we found that the left superior frontal gyrus, the ventral lateral (VL) nucleus of the left thalamus, and the left medial surface of the superior frontal gyrus had the highest likelihood of being activated by familiar music. On the other hand, the left insula, and the right anterior cingulate cortex had the highest likelihood of being activated by unfamiliar music. We had expected limbic structures as top clusters when listening to familiar music. But, instead, music familiarity had a motor pattern of activation. This could reflect an audio-motor synchronization to the rhythm which is more engaging for familiar tunes, and/or a sing-along response in one's mind, anticipating melodic, harmonic progressions, rhythms, timbres, and lyric events in the familiar songs. These data provide evidence for the need for larger neuroimaging studies to understand the neural correlates of music familiarity.

## Introduction

Music is ubiquitous in human culture and has been present since prehistorical times (Conard et al., 2009). Music does not appear to have a survival value, yet most of the current literature has pinpointed it as a fundamental aspect of human life, describing it as a “universal reward” (Trehub et al., 2005). People often value music for the emotions it generates (Juslin and Laukka, 2004; Brattico and Pearce, 2013), and listening to music can help to regulate mood and increase well-being (Hills and Argyle, 1998; Kawakami et al., 2014). This might explain the use of music in people's everyday lives (Schäfer and Sedlmeier, 2010).

Familiarity or repeated exposure in music has been reported as an important factor modulating emotional and hedonic responses in the brain (Pereira et al., 2011). The familiarity principle, also known as the “mere exposure effect,” was first described by Zajonc (1968). It is a psychological phenomenon which suggests that the more exposed we are to someone or something, the more we like it. Repetition in music can be of different types: within a piece, across pieces, or across multiple hearings (Margulis, 2013). Both familiarity and repetition may increase the liking of a piece of music, thus inducing positive emotions (Witviliet and Vrana, 2007; Omar Ali and Peynircioglu, 2010).

Long before its description in 1968, the phenomenon of familiarity had been known by social psychologists and applied to the music field (King and Prior, 2013). The first person who documented it was Meyer in 1903. He presented his subjects with a dozen repetitions of unfamiliar music that he had composed. After listening to the last repetition, most subjects asserted that “the aesthetic effect was improved by hearing the music repeatedly” (Meyer, 1903). Moreover, Meyer showed that melodies which ended on the frequency ratio symbol 2 (the Lipps-Meyer Law) was preferred to all other melodies. However, this law was later on disputed by Paul Farnsworth, his student, who argued that interval ending preferences could be altered by training. Therefore, repetition and familiarity with a specific ratio ending could increase preference for that specific ending. This effect, explaining the perception of music closure, was called the “habit principle” (Farnsworth, 1926). Overall, it seems familiarity deepens the understanding of music and engagement with music listening (King and Prior, 2013).

However, according to numerous studies, the relationship between exposure and enjoyment is non-linear, following an inverted-U shape preference response. Repeated exposure to music can increase pleasure (“hedonic value”) for a certain period, but ultimately gives rise to increasing displeasure (Jakobovits, 1966; Berlyne, 1971; Szpunar et al., 2004; Schellenberg, 2008).

There are different explanations for the inverted U-shape preference response. One is the perceptual fluency model (Bornstein and D'Agostino, 1994) which explains that people incorrectly assume that the facilitated processing of a familiar stimulus is associated to some positive attribute of the stimulus itself. However, as the conscious recognition of fluency processing increases, they stop misattributing this effect to the stimulus but to repeated exposure, and therefore pleasure decreases. Another explanation proposed by Berlyne (1971) states that the inverted U reflects the “interaction of two opposing impulses:” the ascending part arises from an evolutionary conditioned preference for the familiar (positive learned safety effect), and the subsequent decline of the U favors for novelty seeking (aversion to boredom). Moreover, the complexity of the stimulus also influences the timescale of satiation effect. According to Szpunar et al. (2004), despite initial increases in liking, after the stimulus complexity has been absorbed, boredom intercedes, and satiation reduces likability.

Peretz et al. reported that familiarity is best conceptualized as an “implicit memory phenomenon,” in which previous experience aids the performance of a task without conscious awareness of these previous episodes (Peretz et al., 1998). The ability to recognize familiar melodies appeared to be dependent on the integrity of pitch and rhythm perception. Of these two factors, pitch is thought to play a more important role (Hébert and Peretz, 1997). The authors noted that “although the mere exposure effect is simple to define and to reproduce experimentally, it is more complicated to explain.”

Familiarity is a complex subject and the neural mechanisms underlying this memory phenomenon toward music listening are still not very clear or consistent. Some authors define familiarity as a semantic memory process, which is a declarative knowledge (e.g., words, colors, faces, or music) acquired over a lifetime. Musical semantic memory is defined as the long-term storage of songs or musical excerpts, which enables us to have a strong feeling of familiarity when we listen to music (Groussard et al., 2010a). Brain lesion studies showed that music semantic memory appears to involve both hemispheres; however, the integrity of the left hemisphere is critical, suggesting functional asymmetry favoring the left hemisphere for semantic memory (Platel et al., 2003). Neuroimaging studies featuring musical semantic memory have reported the involvement of the anterior part of the temporal lobes, either in the left hemisphere or bilaterally, and the activation of the left inferior frontal gyrus (Brodmann area (BA) 47) (Plailly et al., 2007). Groussard and her co-workers also found activation of the superior temporal gyri (BA 22). The right superior temporal gyrus is mostly involved in the retrieval of perceptual memory traces (information about rhythm and pitch), which are useful for deciding whether or not a melody is familiar. The left superior temporal gyrus seems to be involved in distinguishing between familiar and unfamiliar melodies (Groussard et al., 2010a).

Plailly et al. (2007) also addressed the neural correlates of familiarity and its multimodal nature by studying odors and musical excerpts stimuli. These were used to investigate the feeling of familiarity and unfamiliarity. Results for the feeling of familiarity indicated a bimodal activation pattern in the left hemisphere, specifically the superior and inferior frontal gyri, the precuneus, the angular gyrus, the parahippocampal gyrus, and the hippocampus. On the other hand, the feeling of unfamiliarity (impression of novelty) of odors and music was related to the activation of the right anterior insula (Plailly et al., 2007). Janata (2009) studied the neural correlates of music-evoked autobiographical memories in healthy individuals and those with Alzheimer disease. His findings showed that familiar songs from our own past can trigger emotionally salient episodic memories and that this process is mediated by the medial prefrontal cortex (MPFC). In the same study, hearing familiar songs also activated the pre-supplementary motor area (SMA), left inferior frontal gyrus, bilateral thalamus, and the right cerebellar hemisphere (Janata, 2009).

Brain imaging studies in the neurobiology of reward during music listening demonstrated the involvement of mesolimbic striatal areas, especially the nucleus accumbens (NAcc) in the ventral striatum. This structure is connected with subcortical limbic areas such as the amygdala and hippocampus, insula and anterior cingulate cortex, and also integrated with cortical areas including the orbital cortex and ventromedial prefrontal cortex. These limbic and paralimbic structures are considered the core structures of emotional and reward processing (Koelsch, 2010; Salimpoor et al., 2013; Zatorre and Salimpoor, 2013). Recently, Pereira et al. (2011) investigated familiarity and music preference effects in determining the emotional involvement of the listeners and showed that familiarity with the music contributed more to the recruitment of the limbic and reward centers of the brain.

Electroencephalography (EEG) is another neuroimaging technique that enabled us to address the brain's response to stimuli. It provides a real-time picture of neural activity, recording how it varies millisecond by millisecond. Time-locked EEG activity or event-related potential (ERP) are small voltages generated in the brain structures in response to specific sensory, cognitive or motor event (Luck, 2005). With regards to auditory stimuli—and, more specifically, to music listening and recognition—the N1, P200, P300, and N400 waves have been found to be particularly important. N1, a negative component found 80–110 ms after stimulus onset, is thought to represent the detection of a sound and its features, as well as detection of change of any kind (pitch, loudness, source location etc.) (Näätänen and Picton, 1987; Seppänen et al., 2012). It originates in the temporal lobe, predominantly in or near the primary auditory cortex, suggesting that it is involved in early phases of information processing (Hyde, 1997). Secondly, P2 is a positive component that arises 160–200 ms after the onset of the stimulus (Seppänen et al., 2012) and is localized in the parieto-occipital region (Rozynski and Chen, 2015). It is involved in evaluation and classification of the stimulus (Seppänen et al., 2012) as well as other related cognitive processes, such as working memory and semantic processing (Freunberger et al., 2007). P3, instead, is considered to be more related to selective attention and information processing, such as recognition and memory processes. It is traditionally divided into P3a, arising in the frontal region, and P3b, arising in the temporal and parietal regions; it appears 300–400 ms after the stimulus and lasts 300–600 ms (Patel and Azzam, 2005). However, its timing can vary widely, so it is often described as the late positive complex (LPC), a definition which also includes later deflections, such as P500 and P600 (Finnigan et al., 2002). Finally, N400 arises 200–600 ms after the stimulus, but its anatomical localization has not been well defined since it does not seem to be related to a specific mental operation only. Indeed, it seems to be connected to the processing of meaning at all levels, since it is influenced by factors acting both at lower and at higher levels of these cognitive processes (Kutas and Federmeier, 2011).

Advances in brain imaging techniques have facilitated the examination of music familiarity processing in the human brain. Nevertheless, the use of different modalities and experimental designs has led to differing results. Over the years, studies have used varying music stimuli such as melodies, songs with and without lyrics, with diverse acoustic complexity. Due to this heterogeneity, it is not clear which areas are most reliably engaged when listening to familiar and unfamiliar songs and melodies.

To our knowledge, no systematic review or meta-analysis has been conducted to resolve the inconsistencies in the literature. The present study systematically reviews the existing literature to establish the neural correlates of music familiarity, in healthy population using different neuroimaging methods, including fMRI, PET, EEG, ERP, and MEG. Finally, we used the activation likelihood estimation (ALE) method (Eickhoff et al., 2009) to conduct a series of coordinate-based meta-analyses for fMRI and PET studies. We expected to find brain areas related to emotion or reward as the most active regions when listening to familiar music, as familiarity is positively correlated with likeability and pleasure, at least to a certain number of exposures.

## Materials and methods

### Literature selection

Search Strategy: The search strategy was developed through consultation with the co-authors and a research librarian. The keywords used were (recognition OR familiar OR familiarity OR exposure effect OR repetition) AND (music OR song) AND (brain OR brains OR neuroimaging OR functional Magnetic Resonance Imaging OR Position Emission Tomography OR Electroencephalography OR Event Related Potential OR Magnetoencephalography). The following international electronic databases were searched on July 19th, 2016 and revised on July 11th, 2018: Medline, PsycINFO, Embase. The search was run simultaneously on these databases, using Ovid. For each study included in this review, manual searches of reference lists were conducted for additional articles. Research Ethics Board approval was not required as analysis in this study did not involve data collection.

### Inclusion criteria

Articles selected for inclusion in the systematic review satisfied the following criteria: (a) published between 1996 and 2016; (b) published in English; (c) published in peer-reviewed journals; (d) study results reporting brain regions or coordinates; (e) familiar or unfamiliar music or tone listening as the primary stimulus, regardless of the genre of music and music instrument; (f) sample size to be equal or more than 10 subjects; (g) only experiments with non-clinical adult participants to eliminate potential differences in brain activation that may be associated with neurological or psychiatric illness.

For the meta-analysis the final inclusion criteria were (h) the activation foci where the contrast compared familiar music to unfamiliar music or vice versa; (i) the studies reported whole brain activity, rather than region-of-interest analysis, with complete coordinates of activation in standardized stereotaxic space (i.e., Montreal Neurological Institute [MNI] or Talairach) (Talairach and Tournoux, 1988).

**Figure 1:**
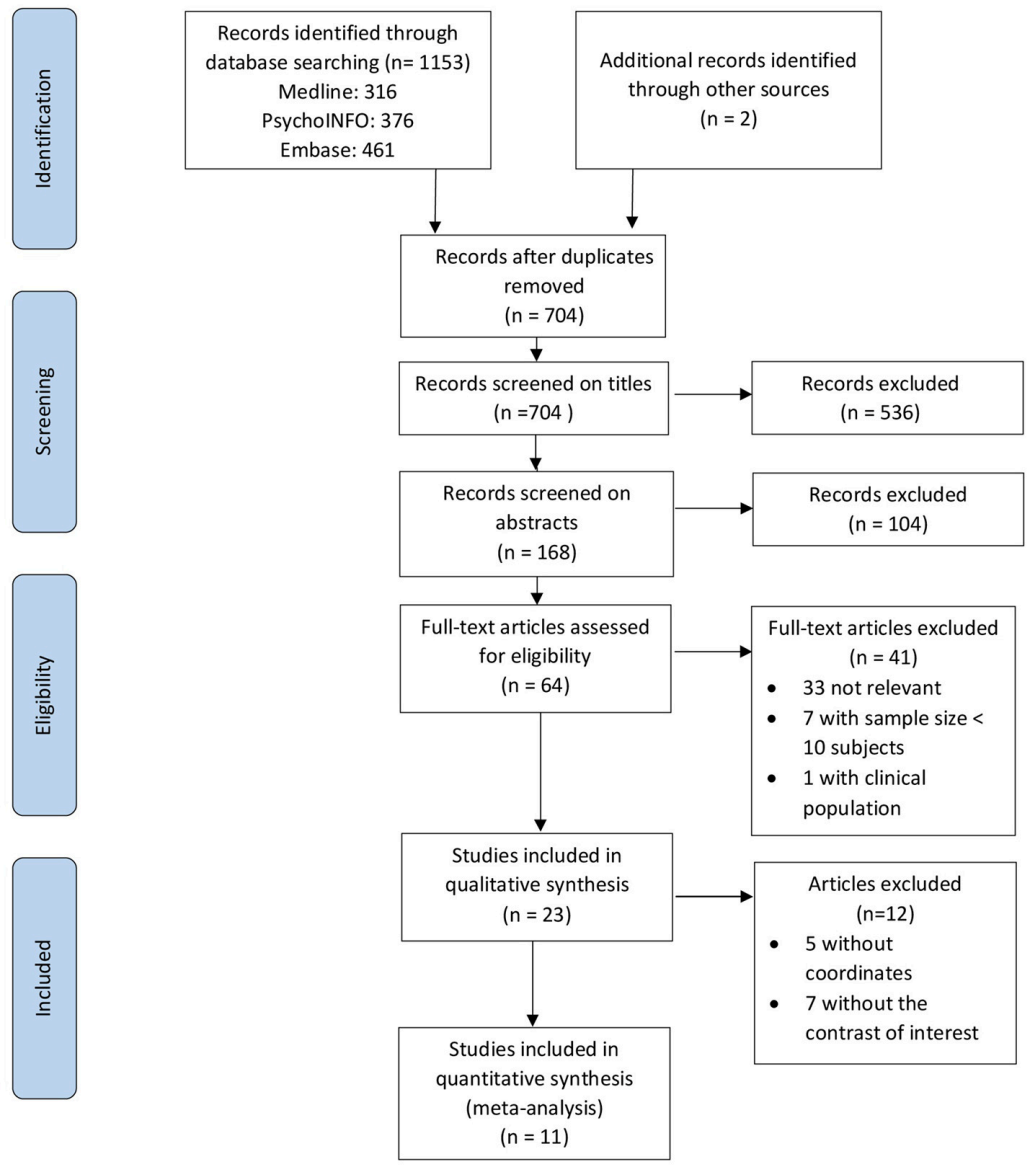
Flowchart of article selection, following PRISMA guidelines. Adapted from Moher et al. (2009).

Two reviewers (CF and EM) independently screened titles, abstracts and full text for relevance. Studies were included if they met the inclusion criteria. We followed the guidelines outlined in the PRISMA (Preferred Reporting Items for Systematic Reviews and Meta-analyses) Statement (Liberati et al., 2009; Moher et al., 2009). The flowchart of article selection is shown in Figure 1.

### Activation likelihood estimation

The meta-analysis was performed using the activation likelihood estimation (ALE) approach (Turkeltaub et al., 2002, 2012; Eickhoff et al., 2009) found in the GingerALE 2.3 software (http://brainmap.org/ale) (Lancaster et al., 2007). As most peak activations (70.0% of all foci) were specified in MNI space, we converted the remaining number of peak activations specified in Talairach space to MNI space using the Brain Map software (http://brainmap.org/icbm2tal/), which implements the Lancaster transformation prior to analysis (Laird et al., 2010). We followed the methodological guidelines described in the user manual for GingerALE 2.3.

We performed independent data-set analyses (one for each contrast), which was used to identify areas of consistent activation across all studies. In the ALE analysis, we first modeled an activation map (MA) for each experiment. These maps were created using the mask, the foci and three-dimensional Gaussian probabilities defined by the full-width at half-maximum (FWHM) values, derived from the subject size (Eickhoff et al., 2009). Subsequently, the union or summation of all MA maps from all experiments produced an ALE map that described the convergence of results at each voxel of the brain, estimating the likelihood of activation at each voxel. Then, the ALE map was compared with an empirically defined null distribution map resulting from a permutation procedure to assess statistical significance (Laird et al., 2009). Lastly, for each meta-analysis, the family-wise error (FWE) method was used to correct for multiple comparisons at a significant cluster level of threshold of *p* < 0.05 (cluster-forming threshold at voxel-level *p* < 0.05, 5000 permutations). However, no peaks survived the correction for multiple comparisons. We also performed an FDR correction which did not reach statistical significance as well. For this reason, we used *p* < 0.001 uncorrected for the threshold, with no minimum cluster size. The use of uncorrected *p*-value threshold is also a valid method described in the user manual for GingerALE 2.3, along with a recommendation to choose a very conservative threshold, such as *p* < 0.001 or *p* < 0.0001. No foci were located outside the GingerALE 2.3 gray matter mask, so there were no foci excluded from analysis.

To visualize the meta-analysis results, ALE output images were overlaid onto an anatomical T1-weighted image, the Colin brain template in MNI space (Holmes et al., 1998). Mango software package version 3.2.3 (http://ric.uthscsa.edu/mango/) was used to create the region-of-interest (ROIs) identified from the ALE analyses. The final ALE scores indicated that the likelihood that any single peak of the total peaks occurred in a single voxel located in the template MRI. These ALE values ranged from 0.007 to a theoretical maximum of 1.0.

## Results

### Results of the literature search

#### Inclusion in the systematic review

Of the 704 titles identified, 23 neuroimaging studies met our inclusion criteria for the systematic review. These consisted of fifteen functional magnetic resonance imaging (fMRI), three positron emission tomography (PET), and five event-related potential (ERP) studies (Table 1). Twelve studies were not included in the meta-analysis for the following reasons: five fMRI (Morrison et al., 2003; Groussard et al., 2010a; Sammler et al., 2010; Herholz et al., 2012; Karmonik et al., 2016) and two PET (Satoh et al., 2006; Saito et al., 2012) did not include the contrast of interest; five ERP studies (Arikan et al., 1999; Zhu et al., 2008; Daltrozzo et al., 2010; Partanen et al., 2013; Chien and Chan, 2015) did not report the coordinates.

**Table 1:** List of the 23 studies, fulfilling the inclusion criteria in the systematic review.

**Year**	**First author**	**No. of participants**	**Age (mean)**	**Control group**	**Method**	**Musical training**	**Inclusion in the meta-analysis or reason for exclusion**
1999	Arikan	10	31	No	ERP	No	Excluded—without coordinates
2003	Morrison	12	34.2	Musicians vs. non musicians	fMRI	Yes	Excluded—without the contrast of interest
2006	Satoh	10	21.6	No	PET	No	Excluded—without the contrast of interest
2007	Plailly	13	27.2	No	fMRI	Yes < 1.5 y	Included
2008	Zhu	15	23	No	ERP	No	Excluded—without coordinates
2008	Nan	20	27.3	No	fMRI	Yes	Included
2008	Watanable	18	22.4	No	fMRI	No	Included
2009	Janata	13	20.0	No	fMRI	n.a	Included
2010	Daltrozzo	21	25	No	ERP	Yes < 1.7 y	Excluded—without coordinates
2009	Klostermann	16	22.4	No	fMRI	No	Included
2010	Demorest	16	28.6	US vs. Turkish	fMRI	Yes < 1 y	Included
2010	Groussard	20	24.5	No	fMRI	No	Excluded—without the contrast of interest
2010	Groussard	20	24.5	No	PET	No	Included
2010	Sammler	12	29	No	fMRI	Yes < 2 y	Excluded—without the contrast of interest
2011	Pereira	15	32	No	fMRI	No	Included
2012	Herholz	10	27	No	fMRI	yes	Excluded—without the contrast of interest
2012	Saito	11	20.8	No	PET	No	Excluded—without the contrast of interest
2013	Partanen	20	4 months	Yes	ERP	No	Excluded—without coordinates
2014	Altenmuller	18	28.7	No	fMRI	Yes	Included
2015	Sikka	40	20; 71	Young vs. old	fMRI	Yes < 3 y	Included
2015	Chien	23	23.1	No	ERP	No	Excluded—without coordinates
2015	Jacobsen	32	28.0	No	fMRI	Yes < 6 y	Included
2016	Karmonik	12	n.a	No	fMRI	Yes	Excluded—without the contrast of interest

*n.a, not available; y, years*.

#### Inclusion in the meta-analysis

Eleven studies (ten fMRI and one PET) involving 212 participants and 145 foci qualified for the meta-analysis using the activation likelihood estimation (ALE) approach (Table 2). The imaging parameters and musical stimuli of included studies are shown in Tables 2, 3, respectively.

**Table 2:** List of the 11 studies, fulfilling the inclusion criteria in the meta-analyses and its imaging parameters.

**Year**	**First author**	**Subjects (N)**	**Method**	**Field strength**	**Imaging sequence**	**Software**	**Blurring kernel**	**Threshold**
2007	Plailly	13	fMRI	3 T	T2* echoplanar	SPM2	7 mm	*P* < 0.01 uncorrected
2008	Nan	20	fMRI	3 T	EPI	LIPSIA	5.65 mm	*P* < 0.001 uncorrected
2008	Watanable	18	fMRI	1.5 T	T2* echoplanar	SPM2	8 mm	*P* < 0.001 uncorrected
2009	Janata	13	fMRI	3 T	EPI	SPM5	5 mm	*P* < 0.001 uncorrected
2009	Klostermann	16	fMRI	4 T	EPI	SPM2	n.a.	*P* < 0.0025 uncorrected
2010	Demorest	16	fMRI	1.5 T	EPI	FSL version 4	5 mm	*P* = 0.05 corrected
2010	Groussard	12	PET	NA	68Ga source	SPM5	12 mm	*P* < 0.001 uncorrected
2011	Pereira	14	fMRI	1.5 T	EPI	FEAT version 5.98	5 mm	*P* = 0.05 corrected
2014	Altenmuller	18	fMRI	3 T	T2* weighted	Brain voyager QX	8 mm	*P* < 0.001 uncorrected
2015	Sikka	40	fMRI	3 T	EPI	SPM 10	8 mm	*P*(FWE)c of 0.05
2015	Jacobsen	32	fMRI	7 T	EPI	SPM 8	n.a.	*P* = 0.001 corrected

*PET, Positron emission tomography; fMRI, functional magnetic resonance imaging; n.a., not available; EPI, Echo planar imaging sequence*.

**Table 3:** Music stimuli characterization (presence or absence of lyrics) of all 11 studies included in the ALE meta-analyses.

**Year**	**First author**	**Method**	**Sample (*N*)**	**Contrast**	**Music stimuli**	**Presence of lyrics**
2007	Plailly	fMRI	13	Familiar music minus unfamiliar Unfamiliar music minus familiar	Instrumental music	No
2008	Nan	fMRI	20	Western vs. Chinese music	Melodies	No
2008	Watanable	fMRI	18	Hits minus CRs	Melodies	No
2009	Janata	fMRI	13	Familiar vs. unfamiliar	Top Pop, R&B songs	Yes
2009	Klostermann	fMRI	16	Hits vs. correct rejections	Musical clips with typical timbre and harmonies	N.A.
2010	Demorest	fMRI	16	Culturally unfamiliar vs. culturally familiar Memory for culturally unfamiliar vs. memory for culturally familiar	Instrumental classic music	No
2010	Groussard	PET	12	Musical semantic > musical reference	Tonal melodies	No
2011	Pereira	fMRI	14	Familiar > unfamiliar Unfamiliar > familiar	Pop-rock songs	Yes
2014	Altenmuller	fMRI	18	Old vs. new pieces New vs. old pieces	Symphonic film music	No
2015	Sikka	fMRI	40	Familiar vs. unfamiliar	Melodies from instrumental pieces	No
2015	Jacobsen	fMRI	32	Long-term known vs. unknown	Top 10 songs from 1977- 2007	Yes

*N.A., not available*.

We identified two contrasts of interest (familiar music minus unfamiliar music and unfamiliar music minus familiar music) and we conducted separate activation likelihood estimation (ALE) meta-analyses for each contrast. Using the ALE approach, we expected to determine the core regions implicated in familiarity and unfamiliarity in music listening.

#### Contrast 1: familiar music minus unfamiliar music

In total, 10 studies (Plailly et al., 2007; Nan et al., 2008; Watanabe et al., 2008; Janata, 2009; Klostermann et al., 2009; Groussard et al., 2010b; Pereira et al., 2011; Altenmüller et al., 2014; Jacobsen et al., 2015; Sikka et al., 2015) were included. This meta-analysis was conducted on 128 activation foci involving 196 participants (Table 4).

**Table 4:** Types of contrasts of the 11 studies included in the meta-analyses.

**Year**	**First author**	**Method**	**Field Strength**	**Subjects (*N*)**	**Contrast**	**Number of foci**	**Type of contrast analysis**
2007	Plailly	fMRI	3 T	13	Familiar music minus unfamiliar Unfamiliar music minus familiar	11 5	1 2
2008	Nan	fMRI	3 T	20	Western vs. Chinese music	10	1
2008	Watanable	fMRI	1.5 T	18	Hits minus CRs	7	1
2009	Janata	fMRI	3 T	13	Familiar vs. unfamiliar	28	1
2009	Klostermann	fMRI	4 T	16	Hits vs. correct rejections	17	1
2010	Demorest	fMRI	1.5 T	16	Culturally unfamiliar vs. culturally familiar Memory for culturally unfamiliar vs memory for culturally familiar	6 1	2 2
2010	Groussard	PET	NA	12	Musical semantic > musical reference	3	1
2011	Pereira	fMRI	1.5 T	14	Familiar > unfamiliar Unfamiliar > familiar	16 4	1 2
2014	Altenmuller	fMRI	3 T	18	Old vs. new pieces New vs. old pieces	2 1	1 2
2015	Sikka	fMRI	3 T	40	Familiar vs. unfamiliar	28	1
2015	Jacobsen	fMRI	7 T	32	Long-term known vs. unknown	6	1

*PET, Positron emission tomography; fMRI, functional magnetic resonance imaging; n.a., not available; CRs, correct rejections; 1 - contrast analysis (familiar music minus unfamiliar music); 2 - contrast analysis (unfamiliar music minus familiar music)*.

#### Contrast 2: unfamiliar music minus familiar music

Four studies (Plailly et al., 2007; Demorest et al., 2010; Pereira et al., 2011; Altenmüller et al., 2014) with a total of 5 experiments, were included. This meta-analysis was conducted on 17 activation foci involving 61 participants (Table 4).

### Results of the ALE meta-analysis

When adopting the threshold for statistical significance corrected for multiple comparisons (using FWE), we did not observe any significant activation for contrast 1 (familiar music minus unfamiliar music), or for contrast 2 (unfamiliar music minus familiar music). We then used uncorrected *P-*value method, but choosing a conservative threshold, *p* < 0.001.

#### Contrast 1: familiar music minus unfamiliar music

**Figure 2:**
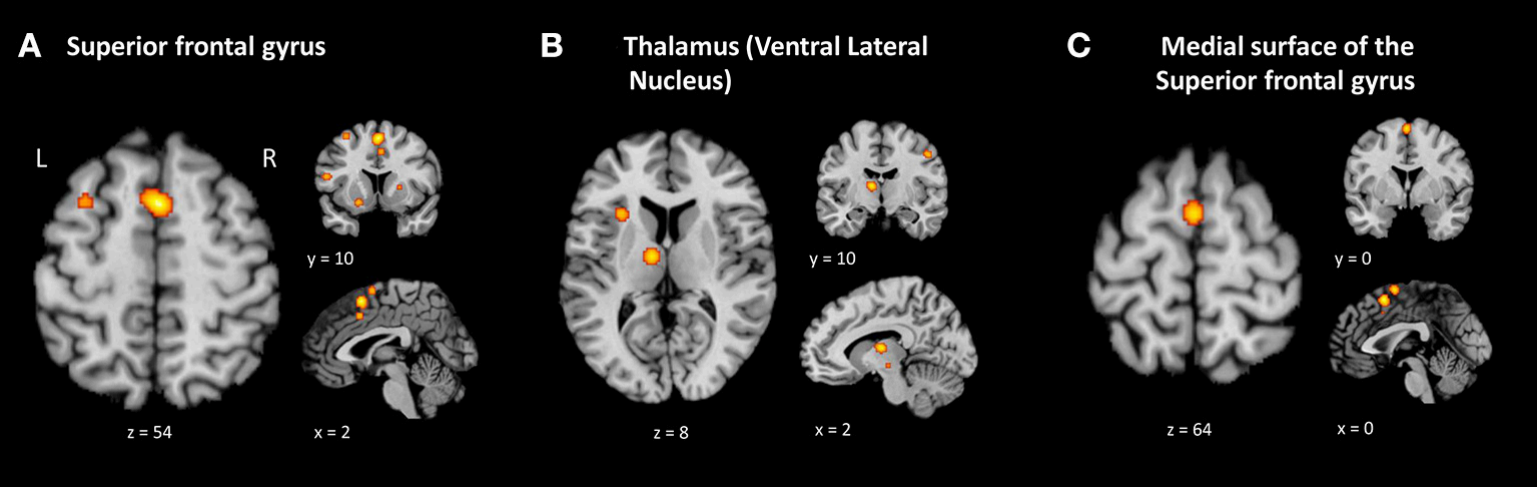
Brain areas showing greater likelihood of activation in familiar music compared to unfamiliar music. ALE maps for the familiar minus unfamiliar music contrast (*p* = 0.001 uncorrected). The three biggest clusters were observed in the left superior frontal gyrus **(A)**, the ventral lateral nucleus of the left thalamus **(B)** and the left medial surface of the superior frontal gyrus **(C)**. Table 5 provides the full list of ALE peaks for this map.

Results of this ALE analysis yielded 37 regions with a significant likelihood (ranging from 0.009 to 0.017) of showing brain activation related to familiarity. The greatest likelihood that activation would be evoked in response to familiar music stimuli was in the left superior frontal gyrus (Brodmann area (BA) 6; ALE = 0.017), the ventral lateral nucleus of the left thalamus (ALE = 0.015), followed by the left medial frontal gyrus, commonly referred to as the medial surface of the superior frontal gyrus (BA 6; ALE = 0.015). A complete list of the ALE values for this study is reported in Table 5 and the top 3 ALE clusters are shown in Figure 2. Supplementary Table 1 displays all contributing studies to each cluster.

**Table 5:** Spatial location and extent of ALE values for contrast 1 (familiar minus unfamiliar music).

**Cluster #**	**Volume (mm3)**	**ALE value**	**MNI**			**Side**	**Anatomical Region**	**BA**	**# of studies contributing to cluster**
			***x***	***y***	***z***				
1	968	0.017	2	10	54	Left	Superior frontal gyrus	6	4/10
2	576	0.015	−10	−10	8	Left	Thalamus (ventral lateral nucleus)	–	3/10
3	440	0.015	0	0	64	Left	Medial surface of the superior frontal gyrus	6	2/10
4	424	0.012	−52	10	14	Left	Inferior frontal gyrus	44	3/10
5	352	0.014	−30	18	6	Left	Claustrum		2/10
6	336	0.012	−52	−42	24	Left	Superior temporal lobe	13	2/10
7	312	0.014	4	12	40	Right	Cingulate gyrus	32	2/10
8	280	0.013	−20	8	−12	Left	Lentiform nucleus. Putamen		2/10
9	280	0.013	50	−8	42	Right	Precentral Gyrus	4	2/10
10	256	0.012	−54	−22	−12	Left	Middle temporal gyrus	21	2/10
11	200	0.012	−4	58	2	Left	Medial frontal Gyrus	10	2/10
12	200	0.012	54	26	32	Right	Middle frontal gyrus	9	2/10
13	192	0.011	8	−26	−2	Right	Thalamus		2/10
14	176	0.011	−32	10	56	Left	Middle frontal gyrus	6	2/10
15	128	0.011	30	−18	−2	Right	Lentiform nucleus.		1/10
16	96	0.010	−42	22	4	Left	Insula	13	1/10
17	64	0.009	22	8	4	Right	Lentiform nucleus		1/10
18	64	0.010	36	42	24	Right	Middle frontal gyrus	9	1/10
19	64	0.009	−26	48	22	Left	Superior frontal gyrus	10	1/10
20	48	0.009	−10	−18	−10	Left	Subthalamic nucleus		1/10
21	40	0.009	−8	12	38	Left	Cingulate Gyrus	32	1/10
22	32	0.008	56	−6	−6	Right	Superior temporal gyrus	22	1/10
23	32	0.008	−32	−14	−4	Left	Lentiform nucleus		1/10
24	32	0.008	10	−8	4	Right	Thalamus		1/10
25	32	0.009	46	20	24	Right	Middle frontal gyrus	9	1/10
26	32	0.008	−50	−6	46	Left	Precentral gyrus	4	1/10
27	16	0.008	40	16	−16	Right	Extra-nuclear	13	1/10
28	16	0.009	−24	26	−8	Left	Claustrum		1/10
29	16	0.009	−4	−24	2	Left	Thalamus		1/10
30	16	0.009	−46	6	4	Left	Precentral gyrus	44	1/10
31	16	0.009	−22	6	4	Left	Lentiform nucleus		1/10
32	16	0.008	−46	26	6	Left	Inferior frontal gyrus	13	None
33	16	0.009	65	−34	14	Right	Superior temporal gyrus	42	1/10
34	16	0.009	−42	6	24	Left	Precentral gyrus	6	1/10
35	16	0.009	52	2	50	Right	Precentral gyrus	6	1/10
36	16	0.009	−44	−4	56	Left	Precentral gyrus	6	1/10
37	8	0.009	−22	52	22	Left	Superior frontal gyrus	10	None

*ALE values for contrast 1. ALE values refer to the likelihood of obtaining activation evoked by listening to familiar music stimuli in a given voxel of the standard template MRI. Coordinates are in the MNI space. Cluster #,The clusters are ranked according to their size in millimeters cubed (mm3). BA, Brodmann area; x, medial-lateral; y, anterior posterior; z, superior-inferior*.

#### Contrast 2: unfamiliar music minus familiar music

**Figure 3:**
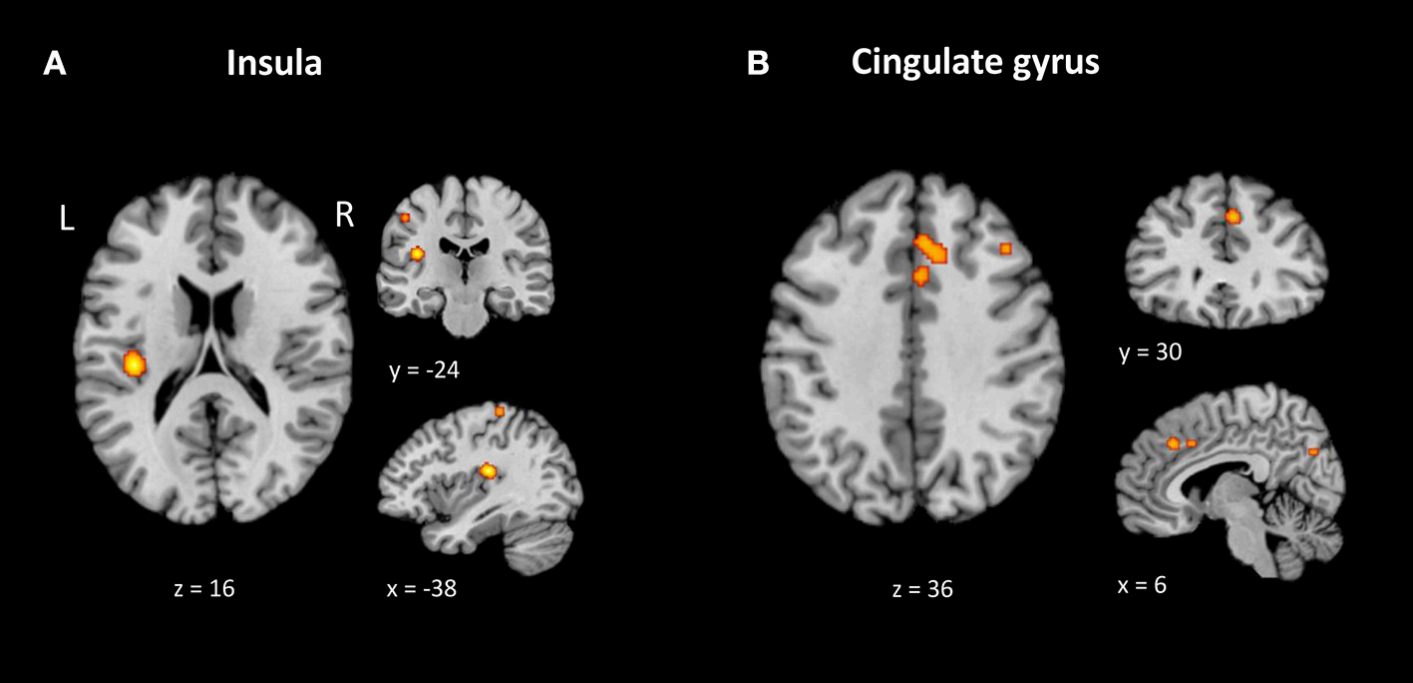
Brain areas showing greater likelihood of activation in unfamiliar music compared to familiar music. ALE maps for the unfamiliar minus familiar music contrast (*p* = 0.001 uncorrected). The three biggest clusters were observed in the left insula **(A)** and on the right cingulate gyrus **(B)**. Table 6 provides the full list of ALE peaks for this map.

The areas with most significant likelihood of activation associated with listening to unfamiliar music were observed in the left insula (BA 13, ALE = 0.012); right cingulate (BA 32, ALE = 0.008 and BA 32, ALE = 0.008) and right middle frontal gyrus (BA 10, ALE = 0.008). All clusters are described in Table 6 and the top 3 ALE clusters are shown in Figure 3. Supplementary Table 2 displays all contributing studies to each cluster.

**Table 6:** Spatial location and extent of ALE values for contrast 2 (unfamiliar minus familiar music).

**Cluster #**	**Volume (mm3)**	**ALE value**	**MNI**			**Side**	**Region**	**BA**	**# of studies contributing to cluster**
			***x***	***y***	***z***				
1	664	0.012	−38	−24	16	Left	Insula	13	2/4
2	488	0.008	6	30	36	Right	Cingulate gyrus	32	2/4
3	176	0.008	4	16	36	Right	Cingulate gyrus	32	$\frac{1}{4}$
4	160	0.008	38	58	−10	Right	Middle frontal gyrus	10	$\frac{1}{4}$
5	160	0.008	8	−72	30	Right	Precuneus	31	$\frac{1}{4}$
6	152	0.008	−42	−78	−4	Left	Inferior occipital gyrus	19	$\frac{1}{4}$
7	152	0.008	16	−92	20	Right	Middle occipital gyrus	18	$\frac{1}{4}$
8	152	0.007	42	−48	40	Right	Inferior parietal lobule	40	$\frac{1}{4}$
9	152	0.007	−48	−24	46	Left	Postcentral gyrus	2	$\frac{1}{4}$
10	152	0.008	−38	−32	62	Left	Postcentral gyrus	40	$\frac{1}{4}$
11	144	0.008	42	58	10	Right	Superior frontal gyrus	10	$\frac{1}{4}$
12	96	0.007	40	−40	32	Right	Supramarginal gyrus	40	$\frac{1}{4}$
13	80	0.007	−28	−18	56	Left	Precentral gyrus	4	$\frac{1}{4}$
14	64	0.007	−21	−75	−47	Left	Inferior semi-lunar lobule		$\frac{1}{4}$
15	64	0.007	41	27	35	Right	Precentral gyrus	9	$\frac{1}{4}$

*ALE values for contrast 2. ALE values refer to the likelihood of obtaining activation evoked by listening to unfamiliar music stimuli in a given voxel of the standard template MRI*.

*Coordinates are in the MNI space. Cluster #, The clusters are ranked according to their size in millimeters cubed (mm3)*.

*BA, Brodmann area; x, medial-lateral; y, anterior posterior; z, superior-inferior*.

### Overview of ERP findings

Our search found five ERP articles, which were published between 1999 and 2015. Regarding the first two studies, Arikan et al. (1999) and Zhu et al. (2008), argued that hearing music of a familiar style increased the allocation of attentional resources during memory updating processes, demonstrated by an increase of P300 (P3) amplitude in frontal areas. Moreover, Zhu et al. (2008) showed a difference in N1 (negative component related to selective attention at an early stage of processing) and later positive complex (LPC; including P300 and P500) between culturally familiar and culturally unfamiliar music.

Daltrozzo et al. (2010) recorded ERPs while participants listened to highly familiar and less familiar melodies in a gating paradigm. The ERPs time-locked to a tone of the melody called the “familiarity emergence point” (defined by Dalla Bella et al. (2003) as the number of tones required for the participant to consider the stimulus as familiar) showed a larger fronto-central negativity for highly familiar compared to less familiar melodies between 200 and 500 ms, with a peak latency around 400 ms. This component was suggested to be N400, a marker of conceptual processing. Overall, this study suggested that the feeling of music familiarity could be accompanied by the processing of the concepts conveyed by emotions to, or semantic associations with, the melody.

Partanen et al. (2013) studied the neural correlates induced by prenatal music exposure using ERPs. After birth and at the age of 4 months, a modified melody was played while ERPs were recorded. Results showed that ERP amplitudes to the changed and unchanged notes of the melody were correlated with the amount of prenatal exposure, suggesting that prenatal exposure to a melody can have long-term plastic effects on the developing brain for at least 4 months.

Finally, Chien and Chan (2015) also concentrated on the N400 wave, but focusing on familiarity effects on the processing of the meaning of lyrics in familiar and unfamiliar songs. Surprisingly, this process is not influenced at all by familiarity, unlike what happens with normal speech. Indeed, repetition usually leads to a decreased processing of meaning (described as “semantic satiation”), but this phenomenon was not observed with song lyrics. Therefore, it would seem that normal speech and lyrics are processed differently, at least at higher levels; in other words, the presence of a melody seems to influence how words are processed.

In sum, ERP studies have suggested an increased attention in frontal brain areas, around 400 ms, when listening to familiar music, and prenatal exposure to a melody can induce neural representations that last for 4 months. Supplementary Table 3 summarizes the outcome measures and main findings of the ERP studies.

## Discussion

To the best of our knowledge, this is the first systematic review and ALE meta-analysis investigating the neural correlates of familiar and unfamiliar music listening. In the following sections we will discuss our findings with respect to familiar and unfamiliar music processing proposals in the literature.

### Overview of ALE meta-analysis findings (fMRI and PET studies)

#### Meta-analysis of activation evoked by familiar music

Literature on “the mere exposure” effect has shown that prior familiarity tends to increase likeability for a stimulus. Moreover, familiarity in music has been reported as an important factor modulating emotional and hedonic responses in the brain (Pereira et al., 2011). For this reason, we expected emotion and reward brain structures to be the top clusters consistently active in the condition of listening to familiar music. To our surprise, the left superior frontal gyrus (BA 6) had the highest likelihood of being activated. This brain area has been previously implicated in the processing of musical semantic memory (Platel, 2005). It may underlie a top-down approach for intentional retrieval of prior episodes or items, selecting them from the semantic memory (Schott et al., 2005; Binder and Desai, 2011).

The ventral lateral (VL) nucleus of the left thalamus had the second highest likelihood of being active in listening to familiar music. This is a motor first-order relay nucleus, which receives input from the substantia nigra, from the internal globus pallidus, and also from the contralateral cerebellum. It also has reciprocal connections with the motor and premotor cortex (Snell, 2010). Three articles (Janata, 2009; Pereira et al., 2011; Altenmüller et al., 2014) contributed to this result. It has been reported by Janata (2009) that the cerebellum is involved in music response planning. Possibly the brain prepares itself to react to music through dance and moves to the beat. Pereira et al. (2011) suggested that, like the putamen, the increased thalamic activity for familiar music could be associated with motor synchronization to the rhythms of the music excerpts, possibly reflecting top-down feedback due to anticipation of the familiar tune. Central thalamus activation seems to regulate attentional resources in task performance, even for very simple tasks, possible through continuing changes in motivation and arousal (Schiff et al., 2013).

As for cluster number 3, the left medial surface of the superior frontal gyrus, it has also been reported in two studies (Pereira et al., 2011; Sikka et al., 2015). This area (*x* = 0: *y* = 0; *z* = 64) can also be labeled as supplementary motor area (SMA) and Brodmann area 6. Pereira et al. (2011) interpreted activations in the SMA by suggesting that the subjects might have mentally sung along with the familiar songs. Halpern and Zatorre (1999) and Halpern (2001) suggested that the SMA is activated during musical imagery, like a sing-along response in one's mind or by anticipating melodic, harmonic progressions, rhythms, timbres, and lyric events in the familiar songs. It is not surprising that passive listening of familiar songs can recruit motor areas of the brain. In fact, auditory and motor systems interact closely during both music perception and production. It has been previously demonstrated that the basal ganglia, cerebellum, dorsal premotor cortex, and SMA are often implicated during music listening (Zatorre et al., 2007; Chen et al., 2008).

Nan et al. (2008) suggested that familiar music could be more appealing than unfamiliar songs and increased attention could be the reason for increased activation in motor areas. Rauschecker (2011) proposed an auditory processing model with an antero-ventral and a postero-dorsal streams. According to this hypothesis, the dorsal stream may play a role in auditory-motor transformations, and the premotor cortex and basal ganglia may be recruited when incoming sounds match expectations based on previous learned ones.

Literature on musical repetition has explored how increased motor activation can aid enjoyment. In her book, *On Repeat*, Margulis (2014) cites Bruno Nettl (1983), an ethnomusicologist who identifies musical repetition as a universal characteristic “shared across music,” (p.19) and Fitch (2006), an evolutionary biologist who calls repetition a “design feature of music” (p.5). Margulis (2014) theorizes that repetition plays a special role in music. As passive music listening recruits motor areas of the brain, Margulis hypothesizes that repeated musical passages are procedurally encoded as chunked automatic sequences, activating motoric basal ganglia. This enhances a listener's ability to automatically anticipate what notes are coming next, without attentional control. As music is repeated and encoded more and more as a fluid sequenced unit, it serves as a hook, compelling a person to execute the sequence imaginatively, without effort. The author suggests that listening to repeated music allows suppression of explicit thought and an increased sense of bodily involvement with the music. Ultimately, this gives a sense of pleasure and transcendence by participation or affiliation with the music (p.12, 67–69, 74).

The notion of musical expectation has been a central issue in music theory, cognition, and aesthetics (Huron, 2006; Huron and Margulis, 2010). Meyer (1956) postulated that expectations play an important role in emotional experiences during music listening. When listening to a musical piece, people can extract implicit, generalized knowledge of musical rules (Tillmann, 2005). This abstract knowledge, also called structural knowledge by Bharucha (1987), allows listeners to create temporal expectancies. The confirmation or violation of the expectancies influences cognitive and emotional experience. Furthermore, anticipation may also arise if one is familiar with the music, and this aspect has been labeled as veridical knowledge by Bharucha (1987).

Taking together, results from previous ERP studies and from this ALE analyses showed that frontal brain areas seemed to be important in the processing of familiar music.

Despite theories demonstrating familiarity increasing pleasurability and liking, there was not much evidence that limbic engagement was modulated by familiarity in this ALE meta-analysis. One possible explanation for this is that ALE analysis is dependent on the coordinates from the original studies, the majority of them did not report limbic structures in their results. Either the music stimuli used were not highly familiar to subjects, or pleasure in music listening was not tied to explicit familiarity.

#### Contrast 2: meta-analysis of activation evoked by unfamiliar music

We explored common brain regions activated by unfamiliar music/tones and found a consistent pattern of activation in the left insula. The insular cortex is associated with cognitive, emotional and regulatory processing, self-awareness and evaluative judgements (Menon and Uddin, 2010; Brattico and Pearce, 2013). The right anterior cingulate cortex (BA 32) had the second and third highest likelihood of being active to unfamiliar music stimuli. This brain area has been implicated in processing emotional salience and motivational aspects of movement (Snell, 2010). Pereira et al. (2011) states that the anterior cingulate cortex has been associated with the judgement of beauty in visual domain studies (Kawabata and Zeki, 2004; Kirk et al., 2009). Other authors, such as Copland et al. (2007) noted that the right anterior cingulate (ACC) is involved in the detection of a prime target relationship. The cingulate gyrus cortex, along with the prefrontal cortex and cuneus, has also been implicated in episodic memory processing for music (Platel et al., 2003; Platel, 2005). In the studies included in this meta-analysis the activation of the ACC might have been associated with successful detection of familiar or unfamiliar song or tones, as subjects had to decide on familiarity. According to Plailly et al. (2007) “the feeling of unfamiliarity refers to the absence of feeling of familiarity.” In sum, the brain regions found to be more active when listening to unfamiliar songs may be related either with the “recognition of the songs or the detection of novelty” (Pereira et al., 2011).

### Limitations and future work

Despite the novel findings of the current study, there are several shortcomings to be addressed.

The first one is that our results lacked significance after correcting for multiple comparisons using FWE and FDR methods and, therefore, are based on conservative but uncorrected p values. As previously mentioned, this is still a valid method described in the GingerALE 2.3 user manual and used in Turkeltaub et al. (2002) study. The second limitation is the small number of studies (n = 11) included in this meta-analysis, limiting the statistical power and sensitivity to detect a common neural mechanism for the listening of familiar music/tones.

The third limitation is related to the statistical robustness of the original studies. Only four of the studies were corrected for multiple comparisons. All other studies reported uncorrected p values (Table 2). We are aware that lenient thresholds (such as *p* < 0.01; *p* < 0.0025 or *p* < 0.001 uncorrected) used in seven of the original studies would have resulted in a larger number of reported foci. In previous versions of GingerALE's methods the number of foci and their proximity of an experiment would determine a greater contribution of that experiment to an ALE map. Consequently, this would give stronger influence to less strict studies (Laird et al., 2005). In version 2.0 GingerALE switched and improved its methods. The modified ALE algorithm eliminated within-experiment effects (Turkeltaub et al., 2012) and incorporated variable uncertainty based on subject size (Eickhoff et al., 2009). As seen in Table 4, the first three studies with greater number of foci included in this meta-analysis used uncorrected p values (please see Table 2). These were Janata (2009), Sikka et al. (2015), and Klostermann et al. (2009). These studies contributed with experiments only for the familiar music minus unfamiliar music contrast and did not have any influence in the meta-analysis of the unfamiliar music. Supplementary Table 1 displays the original studies contributing to each resulting cluster of the ALE method. Janata and Sikka et al. have undoubtedly contributed for the top results of activation evoked by familiar music, but as mentioned above, the sample size in those studies was the weighting factor in the ALE algorithm and not the number of foci.

Fourth, heterogeneity in the type of task and stimuli complexity used across studies may have played an important role in the present results as tasks both with and without lyrics were employed by the original studies. Due to the fact that there were few studies with lyrics within each contrast of interest and consequently lower statistical power, we did not perform separated ALE analyses for studies with and without lyrics. However, we explored whether the overall circuitry would be different if we removed the three studies with lyrics from the ALE analysis. The new ALE analysis eliminated the Superior Frontal Gyrus (a brain region associated with the processing of semantic memory), but the overall brain regions between the studies are highly overlapping, although the order is different. For completeness of reporting, we provide the results table in Supplementary Table 4.

Finally, even though participants in the studies included in the meta-analysis were all non-musicians, half of the studies enrolled participants with musical training. It is known that musical training can change children's brain structure (Hyde et al., 2009). Musicians, compared to non-musicians have better auditory skills, such a larger auditory working memory (Parbery-Clark et al., 2009) and enhanced auditory attention (Strait et al., 2010). Therefore, future studies need to account for stimuli complexity, presence or absence of lyrics, subject characteristics, and music expertise.

## Conclusions

There is a large body of literature highlighting the importance of familiarity and repetition in aesthetics experiences of music. In this study, we have systematically reviewed the literature on the neural correlates of familiarity or repeated exposure of music listening in adult healthy participants. We did not find significant, consistent peak activations among included studies. We had expected limbic structures as top clusters when listening to familiar music. Using a less conservative approach we found, instead, that music familiarity and repetition had a motor pattern of activation.

The implications of this work highlight the need for further larger better-powered studies with more consideration for the nature of the music stimuli and prior music training. The understanding of the neural correlates of music familiarity has the potential to be useful for neurorehabilitation. Future studies involving clinical populations could be optimized and targeted to provide therapeutic support in patients with Alzheimer disease, Down syndrome, and those with severe verbal memory and motor deficits, and language impairments.

## Author contributions

CF, JL, and EA conceived and designed the study. CF, EM, and AB performed the literature selection, data analysis and results interpretation. CF wrote the manuscript. MT, JL, and EA critically revised the work. All authors read and approved the final manuscript.

### Conflict of interest statement

EA has received consultation fees (Roche and Takeda), industry funding (SynapDx and Sanofi-Aventis), royalties (APPI and Springer International Publishing), and editorial honorarium from Wiley. The remaining authors declare that the research was conducted in the absence of any commercial or financial relationships that could be a potential conflict of interest.
