# Supplementary material for: Neural Correlates of Familiarity in Music Listening: A Systematic Review and a Neuroimaging Meta-Analysis
Source: Front Neurosci. 2018 Oct 5;12:686. doi: 10.3389/fnins.2018.00686 (PMC6183416; doi:10.3389/fnins.2018.00686)
Supplement: Supplementary file 1 [file Table_1.DOCX]

Supplementary Material

Neural Correlates of Familiarity in Music Listening: a Systematic Review and a Neuroimaging Meta-Analysis

Carina Freitas^1,2*^, Enrica Manzato ^3^, Alessandra Burini ^3^, Margot J. Taylor ^1,4,5,6^, Jason P. Lerch ^6,7,8^, Evdokia Anagnostou^1, 2, 6, 9^

*** Correspondence:** Carina Freitas: cfreitas@hollandbloorview.ca

# Supplementary Tables

Table 1 – Spatial location and extent of ALE values for contrast 1 (familiar minus unfamiliar music)

| Cluster # | Volume (mm3) | ALE value | MNI | | | Side | Region | BA | Studies contributing  to cluster |
| --- | --- | --- | --- | --- | --- | --- | --- | --- | --- |
|  |  |  | ***x*** | ***y*** | ***z*** |  |  |  |  |
| 1 | 968 | 0.017 | 2 | 10 | 54 | Left | Superior Frontal Gyrus | 6 | 1 focus from Janata  1 focus from Pereira et al.  1 focus from Sikka et al.  1 focus from Jacobsen et al. |
| 2 | 576 | 0.015 | -10 | -10 | 8 | Left | Thalamus (Ventral Lateral Nucleus) |  | 1 focus from Janata  1 focus from Pereira et al.  1 focus from Altenmuller et al. |
| 3 | 440 | 0.015 | 0 | 0 | 64 | Left | Medial surface of Superior Frontal Gyrus | 6 | 1 focus from Pereira et al.  1 focus from Sikka et al. |
| 4 | 424 | 0.012 | -52 | 10 | 14 | Left | Inferior Frontal Gyrus | 44 | 1 focus from Janata  1 focus from Klostermann et al.  1 focus from Sikka et al. |
| 5 | 352 | 0.014 | -30 | 18 | 6 | Left | Claustrum |  | 1 focus from Janata  1 focus from Jacobsen et al. |
| 6 | 336 | 0.012 | -52 | -42 | 24 | Left | Superior Temporal Lobe | 13 | 2 foci from Janata  1 focus from Sikka et al. |
| 7 | 312 | 0.014 | 4 | 12 | 40 | Right | Cingulate Gyrus | 32 | 1 focus from Pereira et al.  1 focus from Sikka et al. |
| 8 | 280 | 0.013 | -20 | 8 | -12 | Left | Lentiform Nucleus. |  | 1 focus from Pereira et al.  1 focus from Sikka et al. |
| 9 | 280 | 0.013 | 50 | -8 | 42 | Right | Precentral Gyrus | 4 | 1 focus from Nan et al.  1 focus from Jacobsen et al. |
| 10 | 256 | 0.012 | -54 | -22 | -12 | Left | Middle Temporal Gyrus | 21 | 1 focus from Plailly et al.  1 focus from Janata |
| 11 | 200 | 0.012 | -4 | 58 | 2 | Left | Medial surface of the Superior Frontal Gyrus | 10 | 1 focus from Plailly et al.  1 focus from Janata |
| 12 | 200 | 0.012 | 54 | 26 | 32 | Right | Middle Frontal Gyrus | 9 | 2 foci from Janata  1 focus from Groussard et al. |
| 13 | 192 | 0.011 | 8 | -26 | -2 | Right | Thalamus |  | 1 focus from Janata  1 focus from Sikka et al. |
| 14 | 176 | 0.011 | -32 | 10 | 56 | Left | Middle Frontal Gyrus | 6 | 1 focus from Plailly et al.  1 focus from Janata |
| 15 | 128 | 0.011 | 30 | -18 | -2 | Right | Lentiform Nucleus. |  | 1 focus from Sikka et al. |
| 16 | 96 | 0.010 | -42 | 22 | 4 | Left | Insula | 13 | 1 focus from Sikka et al. |
| 17 | 64 | 0.009 | 22 | 8 | 4 | Right | Lentiform Nucleus |  | 1 focus from Sikka et al. |
| 18 | 64 | 0.010 | 36 | 42 | 24 | Right | Middle Frontal Gyrus | 9 | 1 focus from Jacobsen et al. |
| 19 | 64 | 0.009 | -26 | 48 | 22 | Left | Superior Frontal Gyrus | 10 | 1 focus from Jacobsen et al. |
| 20 | 48 | 0.009 | -10 | -18 | -10 | Left | Subthalamic Nucleus |  | 1 focus from Sikka et al. |
| 21 | 40 | 0.009 | -8 | 12 | 38 | Left | Cingulate Gyrus | 32 | 1 focus from Sikka et al. |
| 22 | 32 | 0.008 | 56 | -6 | -6 | Right | Superior Temporal Gyrus | 22 | 1 focus from Sikka et al. |
| 23 | 32 | 0.008 | -32 | -14 | -4 | Left | Lentiform Nucleus |  | 1 focus from Sikka et al. |
| 24 | 32 | 0.008 | 10 | -8 | 4 | Right | Thalamus |  | 1 focus from Sikka et al. |
| 25 | 32 | 0.009 | 46 | 20 | 24 | Right | Middle Frontal Gyrus | 9 | 1 focus from Sikka et al. |
| 26 | 32 | 0.008 | -50 | -6 | 46 | Left | Precentral Gyrus | 4 | 1 focus from Sikka et al. |
| 27 | 16 | 0.008 | 40 | 16 | -16 | Right | Extra-Nuclear | 13 | 1 focus from Jacobsen et al. |
| 28 | 16 | 0.009 | -24 | 26 | -8 | Left | Claustrum |  | 1 focus from Sikka et al. |
| 29 | 16 | 0.009 | -4 | -24 | 2 | Left | Thalamus |  | 1 focus from Sikka et al. |
| 30 | 16 | 0.009 | -46 | 6 | 4 | Left | Precentral Gyrus | 44 | 1 focus from Sikka et al. |
| 31 | 16 | 0.009 | -22 | 6 | 4 | Left | Lentiform Nucleus |  | 1 focus from Sikka et al. |
| 32 | 16 | 0.008 | -46 | 26 | 6 | Left | Inferior Frontal Gyrus | 13 | None |
| 33 | 16 | 0.009 | 65 | -34 | 14 | Right | Superior temporal Gyrus | 42 | 1 focus from Sikka et al. |
| 34 | 16 | 0.009 | -42 | 6 | 24 | Left | Precentral Gyrus | 6 | 1 focus from Sikka et al. |
| 35 | 16 | 0.009 | 52 | 2 | 50 | Right | Precentral Gyrus | 6 | 1 focus from Sikka et al. |
| 36 | 16 | 0.009 | -44 | -4 | 56 | Left | Precentral Gyrus | 6 | 1 focus from Sikka et al. |
| 37 | 8 | 0.009 | -22 | 52 | 22 | Left | Superior Frontal Gyrus | 10 | None |

ALE values for Study 1. ALE values refer to the likelihood of obtaining activation evoked by listening to familiar music stimuli in a given voxel of the standard template MRI. Coordinates are in the MNI space. Cluster #: The clusters are ranked according to their size in millimeters cubed (mm3). Abbreviations: BA, Brodmann area; x, medial-lateral; y, anterior posterior; z, superior-inferior.
