# Supplementary material for: Neural Correlates of Familiarity in Music Listening: A Systematic Review and a Neuroimaging Meta-Analysis
Source: Front Neurosci. 2018 Oct 5;12:686. doi: 10.3389/fnins.2018.00686 (PMC6183416; doi:10.3389/fnins.2018.00686)
Supplement: Supplementary file 2 [file Table_2.DOCX]

Supplementary Material

Neural Correlates of Familiarity in Music Listening: a Systematic Review and a Neuroimaging Meta-Analysis

Carina Freitas^1,2*^, Enrica Manzato ^3^, Alessandra Burini ^3^, Margot J. Taylor ^1,4,5,6^, Jason P. Lerch ^6,7,8^, Evdokia Anagnostou^1, 2, 6, 9^

*** Correspondence:** Carina Freitas: cfreitas@hollandbloorview.ca

# Supplementary Tables

Table 2 – Spatial location and extent of ALE values for contrast 2 (unfamiliar minus familiar music)

| Cluster # | Volume (mm3) | ALE value | MNI | | | Side | Region | BA | Studies contributing  to cluster |
| --- | --- | --- | --- | --- | --- | --- | --- | --- | --- |
|  |  |  | ***x*** | ***y*** | ***z*** |  |  |  |  |
| 1 | 664 | 0.012 | -38 | -24 | 16 | Left | Insula | 13 | 1 focus from Plailly et al.  1 focus from Pereira et al. |
| 2 | 488 | 0.008 | 6 | 30 | 36 | Right | Cingulate Gyrus | 32 | 1 focus from Plailly et al.  1 focus from Demorest et al. |
| 3 | 176 | 0.008 | 4 | 16 | 36 | Right | Cingulate Gyrus | 32 | 1 focus from Demorest et al. |
| 4 | 160 | 0.008 | 38 | 58 | -10 | Right | Middle Frontal Gyrus | 10 | 1 focus from Demorest et al. |
| 5 | 160 | 0.008 | 8 | -72 | 30 | Right | Precuneus | 31 | 1 focus from Demorest et al. |
| 6 | 152 | 0.008 | -42 | -78 | -4 | Left | Inferior Occipital Gyrus | 19 | 1 focus from Pereira et al. |
| 7 | 152 | 0.008 | 16 | -92 | 20 | Right | Middle Occipital Gyrus | 18 | 1 focus from Pereira et al. |
| 8 | 152 | 0.007 | 42 | -48 | 40 | Right | Inferior Parietal Lobule | 40 | 1 focus from Demorest et al. |
| 9 | 152 | 0.007 | -48 | -24 | 46 | Left | Postcentral Gyrus | 2 | 1 focus from Plailly et al. |
| 10 | 152 | 0.008 | -38 | -32 | 62 | Left | Postcentral Gyrus | 40 | 1 focus from Pereira et al. |
| 11 | 144 | 0.008 | 42 | 58 | 10 | Right | Superior Frontal Gyrus | 10 | 1 focus from Altenmuller et al. |
| 12 | 96 | 0.007 | 40 | -40 | 32 | Right | Supramarginal Gyrus | 40 | 1 focus from Plailly et al. |
| 13 | 80 | 0.007 | -28 | -18 | 56 | Left | Precentral Gyrus | 4 | 1 focus from Plailly et al. |
| 14 | 64 | 0.007 | -21 | -75 | -47 | Left | Inferior Semi-Lunar Lobule |  | 1 focus from Demorest et al. |
| 15 | 64 | 0.007 | 41 | 27 | 35 | Right | Precentral Gyrus | 9 | 1 focus from Demorest et al. |

ALE values for Study 2. ALE values refer to the likelihood of obtaining activation evoked by listening to unfamiliar music stimuli in a given voxel of the standard template MRI. Coordinates are in the MNI space. Cluster #: The clusters are ranked according to their size in millimeters cubed (mm3). Abbreviations: BA, Brodmann area; x, medial-lateral; y, anterior posterior; z, superior-inferior.
