# Supplementary material for: Neural Correlates of Familiarity in Music Listening: A Systematic Review and a Neuroimaging Meta-Analysis
Source: Front Neurosci. 2018 Oct 5;12:686. doi: 10.3389/fnins.2018.00686 (PMC6183416; doi:10.3389/fnins.2018.00686)
Supplement: Supplementary file 3 [file Table_3.docx]

Supplementary Material

Neural Correlates of Familiarity in Music Listening: a Systematic Review and a Neuroimaging Meta-Analysis

Carina Freitas^1,2*^, Enrica Manzato ^3^, Alessandra Burini ^3^, Margot J. Taylor ^1,4,5,6^, Jason P. Lerch ^6,7,8^, Evdokia Anagnostou^1, 2, 6, 9^

*** Correspondence:** Carina Freitas: cfreitas@hollandbloorview.ca

# Supplementary Tables

Table 3 - List of the ERP studies included in the systematic review

| **Year** | **First author** | **Sample size** | **Age (mean)** | **Type** | **Task**  **Method** | **Outcome measures** | **Main Findings** |
| --- | --- | --- | --- | --- | --- | --- | --- |
| 1999 | Arikan | 10 | 31 | Healthy | ERP | Increase of the P300 (P3) amplitude (reflection of a selective attention and memory updating process) | Hearing music of a familiar style increases allocation of attentional resources |
| 2008 | Zhu | 15 | 23 | Healthy | ERP | P300 amplitude and P500 | Greater P300 amplitude in frontal areas in a culture-familiar music environment |
| 2010 | Daltrozzo | 21 | 25 | Healthy | ERP | Familiarity emergence point | Larger fronto-central negativity N400 for highly familiar compared with less familiar melodies between 200 and 500 ms, with a peak latency around 400 msec. |
| 2013 | Partanen | 20 | 4 months | Healthy | ERP | Positive Mismatch Negativity (MMN) to changed sounds between 200 and 300 ms after stimulus onset | Prenatal exposure to a melody induces neural representations that last for four months |
| 2015 | Chien | 23 | 23.1 | Healthy | ERP | N400 did not vary with subjects’ familiarity with the songs | Repetition in music did not diminish the processing of meaning in lyrics. The presence of melody seems to influence how words are processed. Normal speech and lyrics seem to be processed differently. |
