# Supplementary material for: Neural Correlates of Familiarity in Music Listening: A Systematic Review and a Neuroimaging Meta-Analysis
Source: Front Neurosci. 2018 Oct 5;12:686. doi: 10.3389/fnins.2018.00686 (PMC6183416; doi:10.3389/fnins.2018.00686)
Supplement: Supplementary file 4 [file Table_4.docx]

Supplementary Material

Neural Correlates of Familiarity in Music Listening: a Systematic Review and a Neuroimaging Meta-Analysis

Carina Freitas^1,2*^, Enrica Manzato ^3^, Alessandra Burini ^3^, Margot J. Taylor ^1,4,5,6^, Jason P. Lerch ^6,7,8^, Evdokia Anagnostou^1, 2, 6, 9^

*** Correspondence:** Carina Freitas: cfreitas@hollandbloorview.ca

# Supplementary Tables

Table 4 – Spatial location and extent of ALE values for contrast 1 (familiar minus unfamiliar music) using studies with instrumental music

| Cluster # | Volume (mm3) | ALE value | MNI | | | Side | Region | BA | Studies contributing  to cluster |
| --- | --- | --- | --- | --- | --- | --- | --- | --- | --- |
|  |  |  | ***x*** | ***y*** | ***z*** |  |  |  |  |
| 1 | 288 | .011 | -54 | 10 | 14 | Left | Inferior Frontal Gyrus | 44 | 1 focus from Klostermann et al.  1 focus from Sikka et al. |
| 2 | 200 | .010 | 30 | -18 | -2 | Right | Lentiform Nucleus (Lateral Globus Pallidus) |  | 1 focus from Sikka et al. |
| 3 | 192 | .010 | -42 | 22 | 4 | Left | Insula | 13 | 1 focus from Watanabe et al.  1 focus from Sikka et al. |
| 4 | 80 | .009 | -48 | 6 | 4 | Left | Precentral Gyrus | 44 | 1 focus from Sikka et al. |
| 5 | 64 | .008 | -22 | 8 | -14 | Left | Lentiform Nucleus (Putamen) |  | 1 focus from Sikka et al. |
| 6 | 64 | .008 | 56 | 12 | -14 | Left | Superior Temporal Gyrus | 22 | 1 focus from Sikka et al. |
| 7 | 64 | .009 | -10 | -18 | -10 | Left | Subthalamic nucleus |  | 1 focus from Sikka et al. |
| 8 | 64 | .009 | -4 | -24 | 2 | Left | Thalamus |  | 1 focus from Sikka et al. |
| 9 | 64 | .008 | 50 | 8 | 2 | Right | Precentral Gyrus | 44 | 1 focus from Sikka et al. |
| 10 | 64 | .008 | 40 | 28 | 2 | Right | Inferior Frontal Gyrus | 13 | 1 focus from Sikka et al. |
| 11 | 64 | .009 | -42 | 6 | 24 | Left | Precentral Gyrus | 6 | 1 focus from Sikka et al. |
| 12 | 64 | .009 | 46 | 20 | 24 | Right | Middle Frontal Gyrus | 9 | 1 focus from Sikka et al. |
| 13 | 64 | .009 | 52 | 2 | 50 | Right | Precentral Gyrus | 6 | 1 focus from Sikka et al. |
| 14 | 64 | .009 | -2 | 0 | 64 | Left | Medial surface of Superior Frontal Gyrus | 6 | 1 focus from Sikka et al. |
| 15 | 56 | .009 | -50 | -40 | 24 | Left | Insula | 13 | 1 focus from Sikka et al. |
| 16 | 32 | .008 | 56 | -6 | -6 | Right | Superior Temporal Gyrus | 22 | 1 focus from Sikka et al. |
| 17 | 32 | .008 | 8 | -24 | -4 | Right | Thalamus |  | 1 focus from Sikka et al. |
| 18 | 32 | .008 | -32 | -14 | -4 | Left | Lentiform Nucleus (Putamen) |  | 1 focus from Sikka et al. |
| 19 | 32 | .008 | 8 | -8 | 4 | Right | Thalamus (Medial Dorsal Nucleus) |  | 1 focus from Sikka et al. |
| 20 | 32 | .008 | -8 | 12 | 38 | Left | Cingulate Gyrus | 32 | 1 focus from Sikka et al. |
| 21 | 32 | .008 | -50 | -6 | 46 | Left | Precentral Gyrus | 4 | 1 focus from Sikka et al. |
| 22 | 32 | .008 | -4 | 12 | 54 | Left | Medial surface of Superior Frontal Gyrus | 6 | 1 focus from Sikka et al. |
| 23 | 24 | .009 | -20 | 6 | 4 | Left | Lentiform Nucleus (Putamen) |  | 1 focus from Sikka et al. |
| 24 | 16 | .009 | -24 | 26 | -8 | Left | Claustrum |  | 1 focus from Sikka et al. |
| 25 | 16 | .009 | 22 | 9 | 4 | Right | Lentiform Nucleus (Putamen) |  | 1 focus from Sikka et al. |
| 26 | 16 | .009 | 65 | -34 | 14 | Right | Superior Temporal Gyrus | 42 | 1 focus from Sikka et al. |
| 27 | 16 | .009 | 6 | 14 | 40 | Right | Cingulate Gyrus | 32 | 1 focus from Sikka et al. |
| 28 | 16 | .009 | -44 | -4 | 56 | Left | Precentral Gyrus | 6 | 1 focus from Sikka et al. |
| 29 | 8 | .008 | 36 | -12 | -30 | Right | Parahippocampal Gyrus (hippocampus) |  | 1 focus from Watanabe et al. |

ALE values for contrast 1, using only studies with instrumental music. ALE values refer to the likelihood of obtaining activation evoked by listening to familiar music stimuli in a given voxel of the standard template MRI. Coordinates are in the MNI space. Cluster #: The clusters are ranked according to their size in millimeters cubed (mm3). Abbreviations: BA, Brodmann area; x, medial-lateral; y, anterior posterior; z, superior-inferior.
